# Digital Phenotyping in Livestock Farming

**DOI:** 10.3390/ani11072009

**Published:** 2021-07-05

**Authors:** Suresh Neethirajan, Bas Kemp

**Affiliations:** Adaptation Physiology Group, Department of Animal Sciences, Wageningen University & Research, 6700 AH Wageningen, The Netherlands; bas.kemp@wur.nl

**Keywords:** digital biomarkers, digital phenotyping, wearables, sensors, livestock

## Abstract

**Simple Summary:**

Wearable technology has launched human medicine toward new successes, but are these versatile devices really being leveraged to their best capacity? Applying wearable sensors to animal farming contexts represents tremendous potential for cost-conscious growers and welfare-minded consumers alike. Each farm animal’s phenotype—the set, observable variables that an organism displays based on interactions with their environment—offers unique information on health, welfare, and profitability. Previously, these important observations had to be conducted with extensive time, cost, and labor resources—and even then, the results were impossible to standardize or obtain continuously. Thanks to their proven benefits across many human-based studies, digital phenotype readers can collect and relay specific metrics, such as body temperature, cardiovascular functioning, activity level, and even more complex behaviors such as sociability. Due to cross-species variations, these sensors need to be tailored efficiently and accurately. Future research should inform the design of digital phenotyping options that will offer farmers reliable, robust information, with the long-term goal of creating shared data standards and stores.

**Abstract:**

Currently, large volumes of data are being collected on farms using multimodal sensor technologies. These sensors measure the activity, housing conditions, feed intake, and health of farm animals. With traditional methods, the data from farm animals and their environment can be collected intermittently. However, with the advancement of wearable and non-invasive sensing tools, these measurements can be made in real-time for continuous quantitation relating to clinical biomarkers, resilience indicators, and behavioral predictors. The digital phenotyping of humans has drawn enormous attention recently due to its medical significance, but much research is still needed for the digital phenotyping of farm animals. Implications from human studies show great promise for the application of digital phenotyping technology in modern livestock farming, but these technologies must be directly applied to animals to understand their true capacities. Due to species-specific traits, certain technologies required to assess phenotypes need to be tailored efficiently and accurately. Such devices allow for the collection of information that can better inform farmers on aspects of animal welfare and production that need improvement. By explicitly addressing farm animals’ individual physiological and mental (affective states) needs, sensor-based digital phenotyping has the potential to serve as an effective intervention platform. Future research is warranted for the design and development of digital phenotyping technology platforms that create shared data standards, metrics, and repositories.

## 1. Background

The success and advancement of the livestock industry significantly influence the success of the human population. The human population is expected to expand to 9.7 billion by 2050, placing an increased demand for production on livestock farming methods that have already pushed the limits of efficiency [1]. Confined farming operations have maximized the production of food animals while minimizing space, and other scientific advancements have abbreviated production time. However, the livestock industry also faces growing public awareness of animal welfare and consumer pressures to provide animals with more humane living conditions.

The demands for more livestock production and improved animal welfare seem to contradict [2], but there is a modern solution to both of these pressures—digital phenotyping. This minimally invasive method of data collection has shown promising results in human-based studies while suggesting many applications in the field of animal agriculture. Through the innovations of digital phenotyping, farm animals can be bred, housed, and cared for in ways that improve their production rates, resilience, and well-being. 

This article provides an in-depth survey of the significance of digital phenotyping in relation to farm animals. With continued technological advancements, research, and refined applications, digital phenotyping can improve farming operations and the lives of animals on a global scale. 

### 1.1. Phenotyping

Phenotypes are set, observable variables that an organism displays when interacting with its environment. These variables are influenced by genotypes, which are complex and inheritable. Phenotypes can be measured continuously (e.g., assessing the change in an animal’s body temperature over a day) or categorically through the use of concise scoring systems. When the phenotypes of an animal are measured and collected for use as data points, the process is described as phenotyping [3]. 

Phenotyping has played an important role in assessing mental health concerns, and historically it was conducted through experience-based sampling methods that depended on human compliance [4,5]. In recent years, the challenges posed by constant compliance and active participation have been overcome through the use of digital phenotyping. This technologically advanced method of phenotyping allows for the passive collection of data, which is extremely beneficial in agriculture because farm animals cannot verbally relay their experiences to their caretakers [5]. 

### 1.2. Heritability of a Phenotype

In the agricultural industry, phenotypes represent an important source of information for many reasons, chiefly because they are indispensable in relation to breeding. Because phenotypes are influenced by genotypes, they offer a minimally invasive or non-invasive indicator of an organism’s genetic makeup. By thoroughly understanding phenotypes, the agricultural industry can breed animals with balanced traits related to resilience and production. Research has shown that a multitude of behavioral and physical traits are heritable in animals, including feather pecking in laying hens, immune response in most farm animals, and cannibalism in pigs [3]. If animals are bred to reduce destructive behaviors and promote more relaxed and compatible behaviors, their welfare will improve. It is also interesting to note that, although not all behavioral and medical conditions may be congenital, genetic traits displayed through phenotypes can predict the susceptibility of an organism to certain conditions [6]. 

### 1.3. Biomarkers Associated with Phenotypes

**Figure 1:**
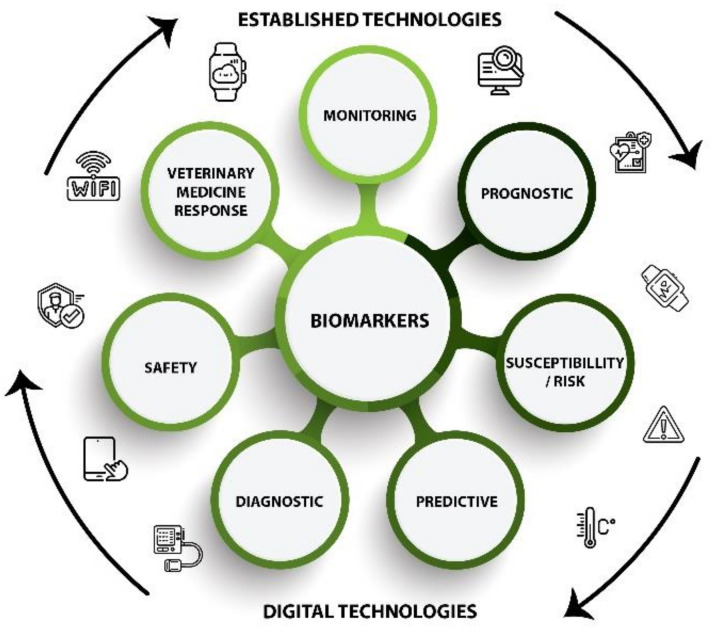
Visual display of digital biomarkers to assess digital phenotyping in farm animals.

Digital phenotyping has been most heavily studied in humans because it provides great opportunities for medical advancement with minimal chances for negative side effects. The phenotypes are chosen and assessed in relation to biomarkers, which are digital measurements of phenotypes (Figure 1) that are categorized according to the intended use of the collected data. The assessment of medical issues in individuals is relatively similar across the spectrum of complex living organisms, especially mammals, which makes these biomarker categories valuable for use within the agricultural industry [7].

To date, the intended purposes of digital biomarkers include the following [7,8]:

- Monitoring = the assessment of a disease, condition, or status that is already known
- Prognostic = the identification of the likelihood of a clinical event occurring/reoccurring
- Susceptibility/Risk = the determination of an individual’s potential to develop a disease or condition with which they are not currently diagnosed
- Predictive = a comparison of similar individuals with different biomarkers to determine divergences in environmental or medicinal effects
- Diagnostic = the detection or confirmation of a disease or condition
- Safety = the assessment of an individual’s fitness in relation to how safely they can engage in certain activities
- Response = the proof of an individual’s biological response to a treatment or environmental change.

When applied to farm animals, these digital biomarkers can help improve research on a multitude of levels, leading to improved animal care on both group and individual levels.

## 2. Impact of Phenotyping in the Livestock Sector

Phenotypes are the basis of generating a more comprehensive understanding of the complexities of raising high-quality livestock. Many aspects of successful agriculture are interrelated and stem from a common point, which is animal welfare. Increased use of antibiotics by farmers for increasing the food safety aspects as part of animal production has devastating consequences for the welfare of animals. Hence, there seems to be an inverse relationship between animal production and animal welfare. However, increasing evidence suggests that animal welfare measures can actually be contributing to sustainable and profitable animal production. Studies have found that animal welfare directly impacts behavior, production capacities, breeding, and disease prevalence [3]. These impacts are generated at an individual level and compounded at a population level. Phenotyping provides specific benefits and opportunities for intervention in each of the aspects listed prior. This allows for better caretaking, especially on a large scale because population shifts are quantifiable through the collection of phenotype-based data points [9]. 

### 2.1. Welfare Implications

Although animals and humans vary in many aspects, they share the need for individual well-being. The multidimensional concept of welfare addresses this universal need by prioritizing the psychological, emotional, behavioral, and physical aspects of an organism’s needs. When animals have each of these needs met, they are most likely to experience a positive sense of well-being among other benefits [3].

The welfare of farmed animals is a necessary but complex topic, as their anthropogenically determined purpose is to produce food, but this often comes at the expense of their lives. Many countries have welfare guidelines that seek to regulate the humane treatment of farm animals, but these guidelines can often fall short of meeting an animal’s actual welfare needs. The only feasible way to create an unbiased assessment of welfare is through phenotyping. Digital phenotyping can assess all of the facets of welfare, even emotional states, which have repeatedly demonstrated accurate measurements from digital phenotyping in humans [5,10,11].

### 2.2. Behavioral Implications

This topic of welfare is highlighted as important to the agricultural industry because animal behavior has a serious impact on production. Animal emotions, which are expressed as aggressive/stress/anxiety/depression related behaviors, have an impact on animal production. The production of farm animals depends not only on the genetic factors and behaviors, but also on animal emotions, which are influenced by several factors including the environment, the relationship with humans and other conspecifics, and the social and emotional development of the animals. Animals with aggressive tendencies toward others can exhibit cannibalistic behavior, guard food sources, endanger caretakers, and cause other disruptive issues within a farm of any scale, especially confined operations. Many behaviors appear to be hereditary, allowing farmers to breed for beneficial behaviors if the target genes are known [3].

In human study subjects, research can assess emotional and behavioral responses with specially constructed digital phenotype assessment profiles [12]. Some of these studies have shown that digital phenotyping can predict emotional and behavioral experiences with up to 95% accuracy [13]. Analyzing sensor data collected from smartphones and wearable sensors using machine learning algorithms, researchers [13] have demonstrated the possibility of predicting depressed mood on an hourly basis. This modeling framework has the potential to be adapted for animal applications as a digital biomarker measurement in measuring behaviors and stress levels. Digital phenotyping enables the development of dynamic models of behavior through empirical data and thereby offers Just-in-Time adaptive interventions, which adjust to an individual animal’s contextual and changing physiological state [14].

### 2.3. Production Implications

The products obtained from farm animals are generally selected for quality and quantity, attributes that may not be identifiable until an animal is at its production peak. At such a point, it may be impossible to breed an animal and pass on its favorable characteristics, making the predictability of production an important consideration. The predictability of actions and outcomes from phenotypic data has been effective in human populations with complex attributes, including physiological states [15]. This information offers great promise in its future application to production-based predictions of livestock.

Niche needs associated with welfare-aligned increases in production can also be met through phenotyping. Studies in humans have demonstrated digital phenotyping to be capable of assessing sleep states [16]. This may seem like pointless information, but for certain animals such as laying hens, assessing the manipulation of circadian rhythms is key to maximizing natural egg production. In a way, phenotyping can facilitate production improvements in livestock by playing on natural functions.

### 2.4. Breeding Implications

Breeding in pursuit of highly productive traits is not equivalent to breeding toward strong reproductive capacities. A prioritization of productivity alone can decrease the resilience and generalized physical fitness of many animals, as demonstrated by the biased or non-data-based breeding of dairy cattle. In contrast, phenotyping provides an opportunity to choose well-rounded breeding animals and to create complementary pairings for stronger future generations [17]. It also helps facilitate breeding interventions at times that maximize fertility and survival.

### 2.5. Disease and Disaster Resilience

Diseases and dramatic natural environmental changes are two factors in farming that are nearly impossible to contain. These factors create a great economic burden and reduce production, but the solution exists within livestock [18]. Their resilience and robustness in the face of these challenges are heritable to some degree and can prepare a population to better face unknown challenges [3]. 

Certain forms of phenotyping have been used effectively to assess the resilience of an organism to pathogenic and environmental stressors, including events such as heat stress [19]. The more resilient members of a livestock group can then be singled out based on their phenotype analysis and used to proliferate their positive traits.

## 3. Overcoming Limitations of Human-Facilitated Livestock Phenotyping

Historically, phenotyping was conducted through personal observations and self-evaluations of human test subjects. These systems allowed for a significant amount of bias between observers and the opportunity for inaccurate evaluations by subjects. To obtain clear and accurate phenotypes, there needs to be an unbiased measurement process that is capable of identifying changes that are otherwise invisible to the human eye [20].

Modern technology has provided a solution to this dilemma in the form of digital phenotyping devices with various biomarker focuses. Not only have many of these devices been comparable if not superior to previous methods, but they also eliminate data-collection bias and reduce the need for stressful human intervention. Furthermore, they collect data at times and in quantities that would be impossible to achieve on a human-facilitated level [21].

### 3.1. Scoring Systems

When phenotypes are only quantifiable through the use of a specially constructed scale or assessment, there is a higher chance of observer bias, especially in the agricultural industry where workers may not share the same regard for all animals [3]. In a review of human biomedical literature, it was found that trained medical professionals could only predict human patient outcomes accurately based on standardized scoring systems 21.4% of the time [22]. 

High standards have been implemented to increase the validity of questionnaire-based scoring systems in human trials, but animals are unable to answer such questions, and their caretakers are unlikely to have the time to fill out these papers on larger farms [23]. Algorithms can be created for the systematic scoring of farm animals based on digitally collected phenotypic traits to create a more modern, efficient, and accurate scoring system [24].

### 3.2. Wearable Sensor Technologies in Phenotyping

Wearable sensor technologies are slowly replacing human-based phenotyping in the medical industry. The capacity of digital biomarkers to offer phenotypic predictions of long-term prognosis using wearable sensors has been demonstrated for predicting anxiety symptoms [25], estimating depression severity [26], and detecting daily life stress [27]. There are plenty of reasons that support this digitally dependent shift, but here are two of the most prominent reasons relating to agriculture.

### 3.3. High-Throughput Phenotyping

High-throughput phenotyping refers to the speeds and quantities at which digital sensor devices can relay data. Human error is a well-known phenomenon in the scientific field, and the most reliable solution is often the addition of automated and technologically advanced elements. These sensors are capable of accurately producing rapid and continuous data points [3]. Their only limits tend to be battery life and range, which are minimal in comparison to the limits of humans attempting to record the same data.

The high rate of data points being produced from both single- and multi-subject studies using these sensors allows valuable data to be fed through specialized algorithms. As with most forms of research and analysis, more data points correlate with improved accuracy when drawing conclusions. In turn, more accurate conclusions can better inform farmers on which changes will offer the greatest benefit to their livestock. 

### 3.4. Assessment of Intersecting Phenotypes

Phenotypes are influenced by a multitude of factors, including environmental changes. When phenotyping data are collected, they are normally observed in association with a certain target, which leaves blind spots for influence by variables that were not apprehended. This event is well explained by a study that sought to determine the cause of fatigue in humans, only to find that the experience of fatigue hinged on diverse phenotypes that were both environmentally and biologically influenced [28]. The method of phenotyping with a specific focus can also influence inaccurate conclusions and actions that lead to trade-offs. In the agricultural field, these trade-offs are most commonly in favor of production but frequently decrease livestock resilience [18].

Wearable sensor technologies can record multiple phenotypes at once, generating more comprehensive datasets. When enough data are collected about phenotypes that may simultaneously change in relation to a single environmental or biological trigger, it becomes possible to determine how they intersect. This information could help farmers avoid breeding practices that result in accidental trade-offs and prevent actions that trigger other unintended results.

## 4. Physiological Measurements by Wearable Sensors for Phenotyping

Sensor technologies are capable of assessing a wide variety of phenotypes. One study even demonstrated the accuracy of a phenotyping technology that assessed diabetes through the analysis of eye phenotypes via photos [29]. Many of the studies supporting the effectiveness of these technologies have been performed on humans, but the results have a high probability of applying to livestock. Humans and animals share many of the same anatomical systems, allowing for the trans-species application of digital phenotyping systems.

**Figure 2:**
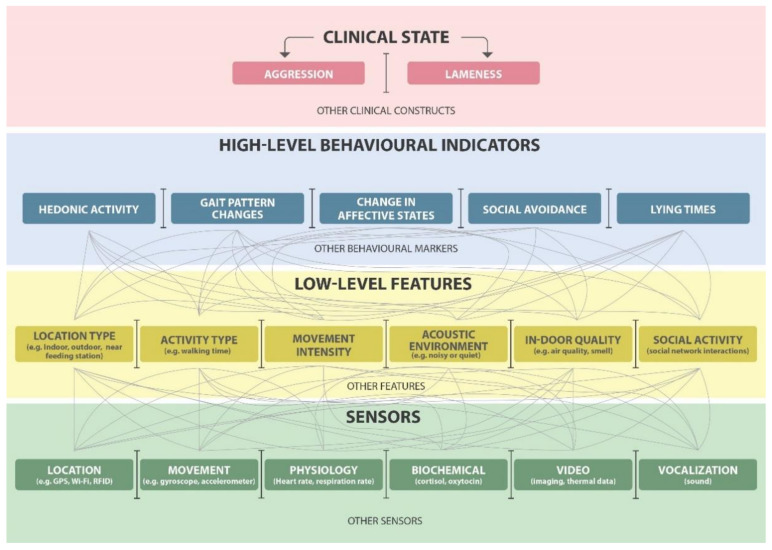
A typical hierarchical and layered sense-making framework for livestock digital phenotyping. Green boxes at the bottom represent inputs of data from sensor platforms; yellow boxes represent the features; blue boxes represent the behavioral biomarkers obtained from the sensors for phenotyping.

These systems may require alterations and advancements for ideal applications to livestock, but they can maintain the same structure of functioning as they do when applied to humans. Figure 2 below illustrates how phenotype sensors can be specified and how their data are analyzed to identify different clinical states [30].

### 4.1. Skin/Outer Body Temperature

Outer body temperatures can vary greatly from an animal’s internal body temperature. This information can help farmers determine an animal’s environmental heat tolerance, a trait that is commonly beneficial in relation to the production of livestock. The following sensors exemplify some of the current methods available for measuring the phenotype of skin temperature through wearable and non-contact technologies. 

#### 4.1.1. Internal Body Temperature

Changes in internal body temperature can signal an imbalance in homeostasis, which is commonly due to heat intolerance or illness. Obtaining an internal body temperature has been an invasive and stressful procedure for animals, as it required the insertion of a thermometer into their rectum on repeated occasions. The digital phenotyping sensors currently being used and developed are considered to be more accurate because they are less likely to cause an increase in body temperature resulting from the unnatural stress of classic phenotyping methods.

#### 4.1.2. Infrared Thermometers

This sensor is also commonly referred to as a heat gun. It uses a fine beam of infrared light to measure the heat being emitted from a surface. This method of measurement is generally accurate, but it can only assess one point of heat dissipation at a time and must be used near the subject [3]. For the phenotyping of small study groups, this measure may be efficient. However, it is not well suited for accuracy when it is applied to many subjects across a large area.

#### 4.1.3. Intestinal Temperature Sensors

Larger livestock, such as cattle and pigs, have been phenotyped for internal body temperatures using intestinal sensors. Because animals enjoy eating, this sensor requires minimal human intervention for insertion. It only needs to be inserted in a bolus and fed to the appropriate animal. These sensors are costly at present because they are new to the world of digital phenotyping, and their recordings can be impacted by the temperature of feed and water [3]. 

#### 4.1.4. Infrared Cameras

Infrared cameras also use infrared light to quantify the amount of heat dissipation from an animal. However, this sensor can generate accurate readings from greater distances and provide a full image of an animal’s heat dissipation profile [3]. A continuous stream of information can be combined with other phenotypic sensors to assess an animal’s location and social interactions all at once. Most importantly, animals are unlikely to notice this form of phenotypic sensor, which reduces the risk of observer influence. 

#### 4.1.5. Contact Sensors

Unlike the two previous sensors, a contact sensor must be attached to the skin of an animal to provide accurate temperature readings. Studies have suggested that this form of outer body temperature measurement is most accurate, but it still has drawbacks, especially when applied to agricultural situations. The two most pronounced complications are the limitations of battery life and the ability of this sensor to adhere to the skin without causing damage or facilitating premature removal [3]. With some animal-specific alterations, this method of digital phenotyping could be far more useful and combined with some of the other sensor systems that will be discussed. 

#### 4.1.6. Surgical Implants

Surgical implants offer continuous data collection at preset intervals. They are nearly impossible for an animal to lose and provide accurate internal temperature readings without further human intervention. The drawbacks of this method include a risk of infection after insertion and the need to wait until the site is fully healed to collect data [3]. 

#### 4.1.7. Inter-Vaginal Temperature Devices

This form of internal body temperature recording is also known as a thermochron temperature recorder and is only applicable to female animals. However, it is considered to be highly accurate, and the data points that it collects can be transferred farther than the intestinal sensor method [3].

#### 4.1.8. Ear Canal Radio Telemetry

Ear canal sensors are currently specialized for use in cattle because they attach similarly to identification ear tags. These sensors provide distanced data transfer and can be theoretically applied to pigs as well [3]. The primary drawback is the tendency for this sensor to fall off because it is mostly exposed, unlike the other three body heat sensors described earlier.

### 4.2. Cardiovascular Health

The cardiovascular system is an integral part of all animals. Research has linked its poor function to multiple disorders of both the mind and body [31]. Insight into the pulse rate of an animal also demonstrates stress levels in response to various environmental stimuli. Furthermore, data related to cardiovascular function can help identify animals with increased longevity and favorable resilience to stressful events [32].

#### 4.2.1. Echocardiogram Imaging

Cardiovascular health is often assessed through blood studies and other invasive procedures that can be especially stressful for animals. Studies in humans have determined that echocardiograms, which do not require phlebotomy, are capable of determining abnormalities in blood qualities such as hemoglobin, blood urea nitrogen, and other helpful markers of medical conditions [33]. If applied to animals, this observation method could offer a functional alternative to blood sampling that provides visual phenotypes of the cardiovascular system as well. 

#### 4.2.2. Photoplethysmography

This method of cardiac health assessment is managed by a small phenotyping device that measures pulses through non-invasive skin contact. This sensor is commonly used in human studies, where it has accurately predicted the presence of diabetes and the onset of stress [34,35]. An animal-tailored variation would provide a minimally influential way to collect daily data related to health and welfare on farms.

### 4.3. Sweating Rate and Composition

Sweat is a form of moisture loss that is directly associated with heat tolerance. The composition of sweat also explains physiological processes happening within an animal through the compounds it contains, such as lactate, an indicator of physical stress [19,36].

#### Digital Moisture Sensors or Galvanic Skin Response

Sweat can be collected in a non-invasive manner; with the proper technology, minimal human interventions are needed. Digital moisture sensors are a type of dermal technology that can relay information about the amount of sweat produced and its components. Human studies have shown that this form of digital phenotyping can determine minute changes that may otherwise be missed. Changes in sweat gland activity have been linked to both emotional and physical arousal in humans [37,38]. These findings suggest that, with a proper understanding of species-specific responses, animal-based applications are feasible [3].

### 4.4. Dietary Health by Ingestible Sensors

Livestock animals’ diets and intestinal health can maximize or seriously impede their productivity. On farms with numerous animals, it can be difficult and even impossible to determine how often, much, and well each animal is eating. The solution can be found in sensors that continuously collect data and feed the results to an analyzing program. The findings could guide welfare changes by indicating the specific needs of the animals being studied. Through monitoring the feeding pattern and the behavior and the associated deviations with the aid of sensor technologies, subclinical health issues and welfare states of farm animals can be identified [39,40].

#### 4.4.1. Bolus Devices

Bolus sensors were noted earlier for their ability to be used as minimally invasive internal body temperature recording devices. They appear also under the topic of dietary health due to their ability to remain in the digestive systems of cattle and pigs for extended periods without negative impacts. Bolus sensors can monitor pH and metabolic status and record nutrition absorption data [3]. All of these factors help determine the health of an animal’s intestinal tract. In cattle, rumen microbe compositions are hereditary to some extent and impact health and production, making them a phenotype worth assessing [17].

#### 4.4.2. Feed-Intake Assessment Systems

Livestock feed is normally dispensed for consumption by large groups, but the addition of feed-intake assessment systems can record individualized data related to feeding frequency, duration, and location. All of these factors relate to the quality of social interactions, disease resilience, and other aspects of animal welfare [3]. In most cases, these sensor-based assessment systems are directed at dairy cattle, consisting of single-spaced feeders that weigh the food and identify individual animals through radio-frequency signals from associated tags. The crosstalk between the animal’s collar sensor and the feed intake sensor of the feed bins helps to monitor the frequency and amount of feed intake and the nutrition involved in the feed, and thereby can inform the health- and productivity-associated indicators through metabolomics [41].

### 4.5. Respiratory Health

Most livestock animals are maintained in group housing to maximize production in minimal space. When implemented properly, this method of agriculture can be high-yield. However, close proximity leaves animals susceptible to contagious diseases, including respiratory ailments. Early identification of respiratory disease is key to containment and treatment, which are significant reasons to employ respiratory phenotyping sensors.

#### 4.5.1. Breath Analysis

Various respiratory diseases spread via airborne transmission. Theoretically, this would make the diseases identifiable through the breath analysis of afflicted animals. Common livestock respiratory viruses that pose great financial and welfare concerns include bovine respiratory disease, tuberculosis, and brucellosis [3]. The early identification of these diseases through digital respiratory analysis could trigger early intervention to minimize impact and help farmers identify the most resistant animals on their farms to breed for stronger immunity.

#### 4.5.2. Auditory Disease Identification

Many respiratory diseases cause distinct coughs and other respiratory changes, depending on their host. In a human subject study, auditory devices were capable of accurately distinguishing breathing patterns and coughs caused by asthmatic episodes [42]. This information would suggest that the same method of digital audio analysis could identify certain respiratory diseases when applied to animals.

### 4.6. Sleep Quality

Sleep and resting play a crucial role in the development and health of most animals. Sleep quality of farm animals is not influenced only by genetic factors but also by the physical and social environments. Some studies have found that inefficient sleep patterns can shorten the telomeres on an animal’s chromosomes, leading to shorter lifespans [43]. This important factor for production is not currently observable for all livestock, and it cannot be easily monitored by human caretakers without the risk of observer influence. Thus, the ability to assess digital phenotypes that can flag sleeping issues can help farmers make adjustments to improve their animals’ health and well-being. 

#### Heart-Rate Measuring Devices

Sleep patterns have been studied in humans through the use of digital phenotyping sensors focused on heart rate and activity [43]. These two points of focus can provide insight into many aspects of an animal’s life, offering a multifaceted tool when properly altered to suit the target species.

### 4.7. Activity Sensors

Activity sensors commonly include accelerometers, pedometers, and GPS devices that can observe location, speed, and time of movement. The activity of an animal can highlight its social interactions and psychological state. In studies of chickens, researchers identified the onset of feather pecking and preferred laying spaces through the use of activity sensors [3]. For humans, a similar device was shown to predict episodes of high anxiety with great accuracy [44]. These sensors do not require constant human intervention and can be fastened to animals in non-invasive ways. These two points are significant because they decrease the chances that the data collected will be unnaturally influenced, a problem that has been noted with many artificially constructed human social activity-tracking studies [45]. In some human-subject trials, activity sensors have even been able to sense body positioning [46]. This valuable information can be collected continuously, providing insight into the progression or decline of an animal’s mobility as influenced by genetics or outside factors [3]. The pedometer has been shown to be a reliable tool for estrus detection through measurement of lying behavior and rumination time in dairy cows [47]. With specialized devices, activity sensors could pinpoint areas of poor mobility in specific individuals, encouraging proper treatment and balanced breeding.

### 4.8. Behavior

Generally, the behaviors of livestock result from their genetics, environment, and experiences. Certain behaviors can be destructive and dangerous to both a single animal and others nearby, while other behaviors can have positive impacts (e.g., easier handling). Often, behavior is interpreted through a lens of human bias, making digital measures more accurate in their potential influence on breeding and groupings of animals.

#### Thermal Imaging

Thermal imaging cameras were discussed earlier as a productive method for assessing the external body temperature of animals. They can also serve as a valuable tool for recording data related to social behavior. Thermal cameras allow for the clear identification of animals, even when they overlap in physical space [3]. Digital cameras and infrared cameras can also be used to record phenotypes related to how animals interact with each other and their caretakers. Cameras can even be used in combination with audio cues, such as crying young, to determine if an animal will show favorable maternal traits [48]. 

### 4.9. Acoustic Sensors

Sound-based data sensors can be attached to individual animals for sound analysis. As demonstrated by human studies, acoustic sensor data can be refined and analyzed to predict emotional states and some actions [49,50]. It is not unreasonable to expect the same to be true for animals due to their abundant vocalizations that are specific to stress, breeding, eating, and socialization. The only major problem with this digital phenotyping device is refining its ability to distinguish the noises of specific individual animals in crowded agricultural settings [3]. 

### 4.10. Light Sensors

For humans, receiving an appropriate amount of sunlight supports proper mental and physical health [51]. Although only some farming operations allow outdoor spaces for their animals, natural light detectors could still be useful sensors. For example, their data could help determine which animals are most comfortable in outdoor spaces by tracking their behavior-related phenotypes in relation to sun exposure. Animals who prefer to be outside even on hotter days could be exemplifying greater heat tolerance and robustness.

### 4.11. Mobility, Posture, and Lameness

Aspects of mobility are important for all humanely raised farm animals, as they often have to rely on movement for eating, drinking, breeding, and other activities related to welfare. Animals with poor physical conformations are prone to lameness, a painful experience that can lead to premature death. In some aspects of farming, animals will not be accepted for slaughter if they cannot walk into the facility independently. Other times, herd lameness raises great welfare concerns and causes decreases in production. Many components of mobility are hereditary and environmentally driven, and proper phenotyping could facilitate the genetic advancement of farm animal mobility.

### 4.12. Cameras

Cameras of all types can serve as an effective measure for assessing mobility. Depending on where they are mounted in a facility, these devices can provide clear views of animals’ locomotion in positions that human observers cannot safely reach. Cameras also allow for continuous observation of quantifiable mobility phenotypes for specific animals [3]. In comparison, human observers on most farms may only spend a few minutes observing each animal, indicating their phenotyping has a greater possibility for bias. With the 3D motion learning artificial learning techniques, cameras have the potential to overcome barriers in measuring animal activity and postures, varying lighting conditions, and occlusions. However, these advancements are only at the preliminary research stage and have not yet found practical applications in commercial settings. Using cameras and the analysis of images of facial features of cows and pigs, researchers [52] were able to measure inferred emotions and determine whether the animals displayed aggression or excitement or neutral affective states. The ability to determine emotions of farm animals using cameras provides ways to enhance animal–human interactions and animal welfare.

## 5. New Phenotyping Platforms for Accurate Assessment of Livestock

**Figure 3:**
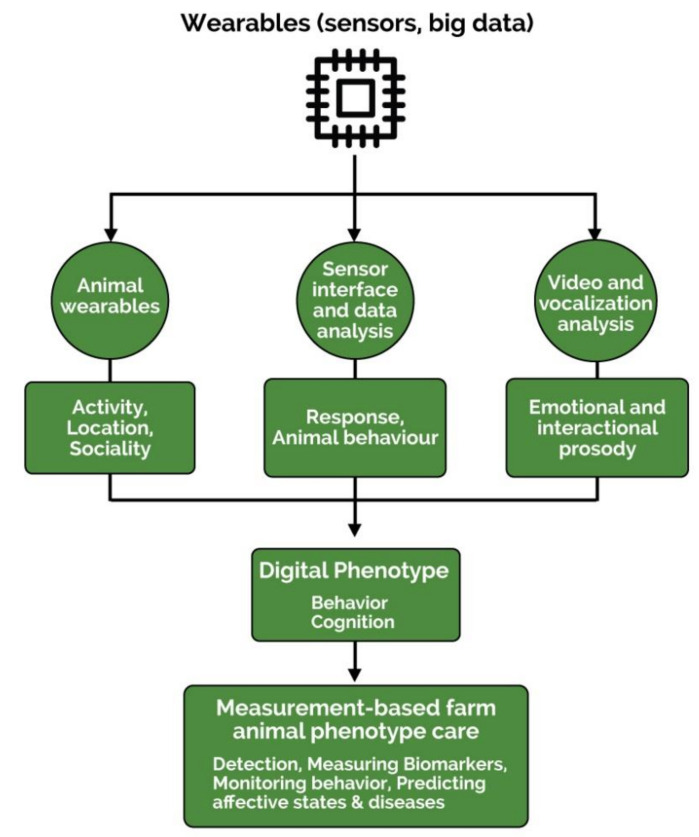
Digital phenotyping of farm animals involves collection of data from animal wearables and video and sound-based data to measure behavior and predict disease and cognition.

The field of digital phenotyping is expected to continue its evolution toward more precise and compatible sensor systems, especially in the field of human medicine. This is a promising step in the trickle-down of new technology for the agricultural industry, but with a growing human population and public pressures, it is unreasonable to wait for the simple application of human-tested sensors for farm animals. Instead, it would be in the agricultural industry’s best interest to start testing, refining, and creating digital phenotyping sensors that are specific to livestock species. The sensor systems described above are all promising technologies, but many of them are based on human models (Figure 3) [22]. 

This image depicts the sensor sequence commonly used to assess phenotypes in humans. The sequence from a device to measurement-based care represents a valid application to livestock. However, device and sensor foci must be altered for accurate use in animals. Research has suggested that improvements and alterations of digital phenotyping devices can be managed most appropriately through the acceptance of an open interface. This structure will allow for infinite growth of technology in whatever direction is needed for a given species because digital phenotyping is a dynamic field of study [8]. With proper information protection, the acceptance of a shared data system could also improve efficiency, standardization, and welfare, much as it has for hospital data systems [12,53]. This shared information could help catalyze evaluations of geographical or global complications within a species, and it even opens up the opportunity for genetically diverse and data-backed breeding programs. 

Other studies have highlighted the importance of making digital phenotyping a more accessible and understandable form of measurement to improve its usage [54]. This suggestion can be extremely beneficial in the realm of farming, as an accessible digital phenotyping system could help a multitude of farmers make educated animal care decisions without extra schooling. Accessibility also includes affordability, a factor that will hopefully increase with the development of tailored systems. Some studies have found accurate values from sensors that only take hourly readings, as opposed to continuous readings [36]. This method could increase affordability by saving battery power and data storage space.

Many other minute alterations are needed for the advancement of phenotyping technologies, but automated interventions based on phenotypic assessments are one of the most interesting and impactful concepts being studied [55]. Frequently, the data-collection systems analyze information relayed from digital sensors and generate diagnostic results, but this pathway could be taken a step further. For example, the automation of a systemic response to specific diagnostic findings could help provide immediate care to farm animals while simultaneously reducing the need for manual labor. However, this concept still requires extensive research and testing, even in human-based studies. 

Digital phenotyping is basically quantitation of farm animals’ in situ phenotypes using individual wearable sensors or through video image analysis. The sensors that have been used so far on animals in the livestock sector are primarily geared toward measurement of physiological and behavioral parameters from a precision livestock farming context. From an instrumentation and sensor engineering perspective, it would not be practically feasible to provide "efficacies" of the methods for each and every individual sensor type, due to the context-based measures in various farm animal applications and the cross-sensitivity and specificity issues in real-time deployment. An example would be the application of camera sensors to measure the activity of chickens. The efficacy of cameras in determining the activity depends on lighting conditions, the height or position of the camera deployed, occlusions and other visible barriers present on the farm, and camera hardware parameters determining the resolution etc. The efficacy of camera-based systems will vary greatly between species such as chicken vs. dairy cow vs. pig movement and activity.

Current research advancements are focused on the development of multiplexed, multifunctional sensors that can measure heterogeneous phenotyping data from a single device. Data fusion technology provides an enhanced way of measuring parameters with much accuracy and information richness in the precision livestock digital phenotyping field. The deployment of multifunctional sensors in livestock animal farming will overcome the wearable factor, occupying less space and ensuring skin conformability with reductions in the cost of manufacturing. However, reliability and sensor data fusion related issues still need to be overcome. With the advent of artificial intelligence and sensor technology advancements, data fusion technology will become the basis for real-time digital phenotyping.

## 6. Summary

Digital phenotyping represents a beneficial opportunity with several complexities. Namely, it can provide an extrinsic way of enhancing the knowledge and prediction of resilience and disease-indicating factors among farm animals, but it also further removes any relationship between animals and caretakers [56]. Furthermore, digital phenotyping can only complement human interventions and cannot offer a causal explanation for the data collected. Despite these significant considerations, digital phenotyping for livestock represents an emerging direction of research and potential application efforts that are both scientifically intriguing and practically warranted.

## Data Availability

Not applicable.
